# RETRACTION: Engineered IL‐7 Receptor Enhances the Therapeutic Effect of AXL‐CAR‐T Cells on Triple‐Negative Breast Cancer

**DOI:** 10.1155/bmri/9758091

**Published:** 2026-10-07

**Authors:** BioMed Research International

RETRACTION: Z. Zhao, Y. Li, W. Liu, X. Li, “Engineered IL‐7 Receptor Enhances the Therapeutic Effect of AXL‐CAR‐T Cells on Triple‐Negative Breast Cancer,” *BioMed Research International*, 2020, 4795171, https://doi.org/10.1155/2020/4795171

The above article, published online on 08 January 2020 in Wiley Online Library (wileyonlinelibrary.com), has been retracted by agreement between the journal Editor‐in‐Chief, Yoel Klug; and John Wiley & Sons Ltd. The retraction has been agreed due to concerns reported for Figure 4, both directly and on PubPeer [1]. Specifically:

• In Figure 4a, the Day 14/AXL‐CAR.C7R panel appears highly similar to the Day 21/AXL‐CAR panel.
• In Figure 4a, the Day 21/AXL‐CAR.C7R panel appears highly similar to the third panel down in Figure 4c of [2]
• In Figure 4a, the Day 21/C7R panel appears highly similar to the fifth panel down in Figure 4c of [2]
• In Figure 4a, the Day 28/PBS panel appears highly similar to the seventh panel down in Figure 4c of [2]
• In Figure 4a, the Day 28/C7R panel appears highly similar to the eighth panel down in Figure 4c of [2]

The authors were unresponsive when asked for clarification of the above, and after assessment by the Chief Editor, the results and conclusions can no longer be considered reliable. The article is therefore retracted.

The authors did not respond to the retraction.
